# Supplementary material for: The Gut-Ex-Vivo System (GEVS) Is a Dynamic and Versatile Tool for the Study of DNBS-Induced IBD in BALB/C and C57BL/6 Mice, Highlighting the Protective Role of Probiotics
Source: Biology (Basel). 2022 Oct 27;11(11):1574. doi: 10.3390/biology11111574 (PMC9687175; doi:10.3390/biology11111574)
Supplement: Supplementary file 1 [file biology-11-01574-s001.zip › biology-1957560-supplementary.pdf]

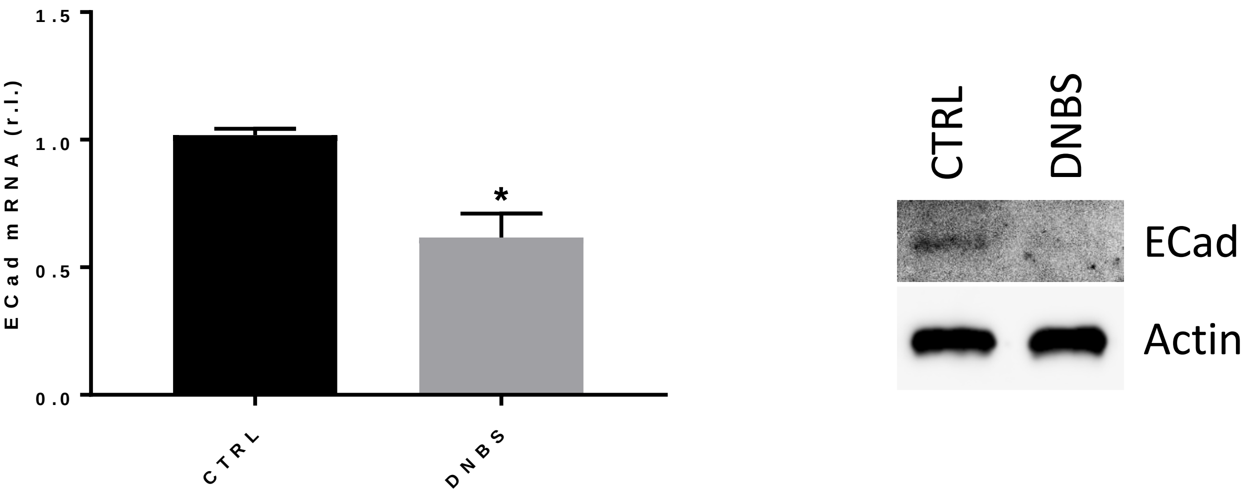

Figure S1. E-Cadherin expression modulation by DNBS. Colon from C57BL/6 mice were untreated (CTRL) or exposed to DNBS (1,5mg/ml; B) for 5h, in GEVS, and the expression of E-Cadherin was evaluated by both RT-qPCR (A) and western blotting (B) analysis. Actin was used as loading control. Data are representative of experiments performed three times, in triplicate. Histograms are representative of mean ± standard deviation; * p < 0,05.

A

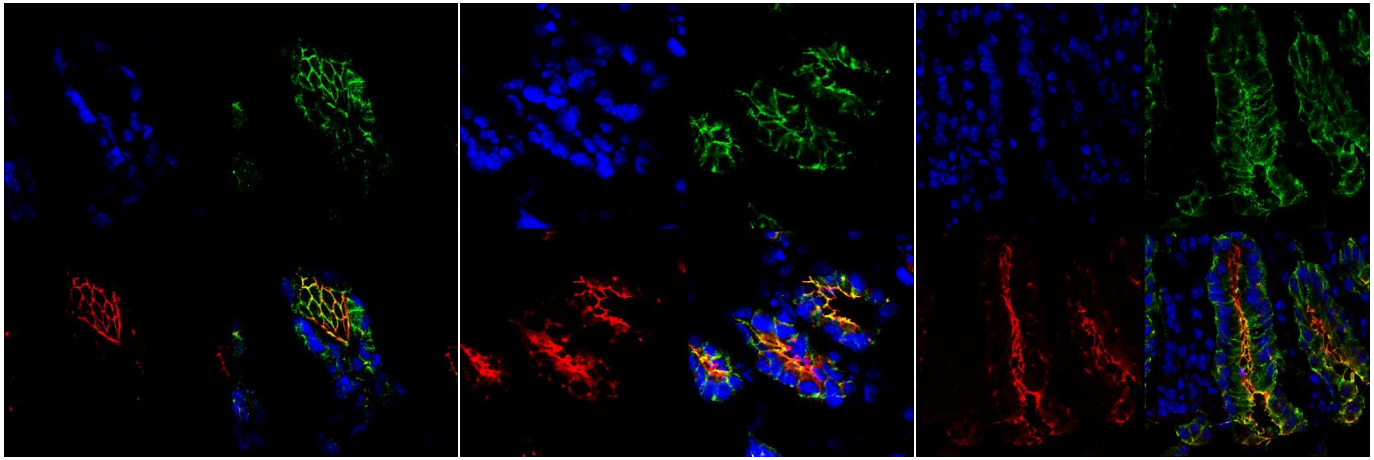

B

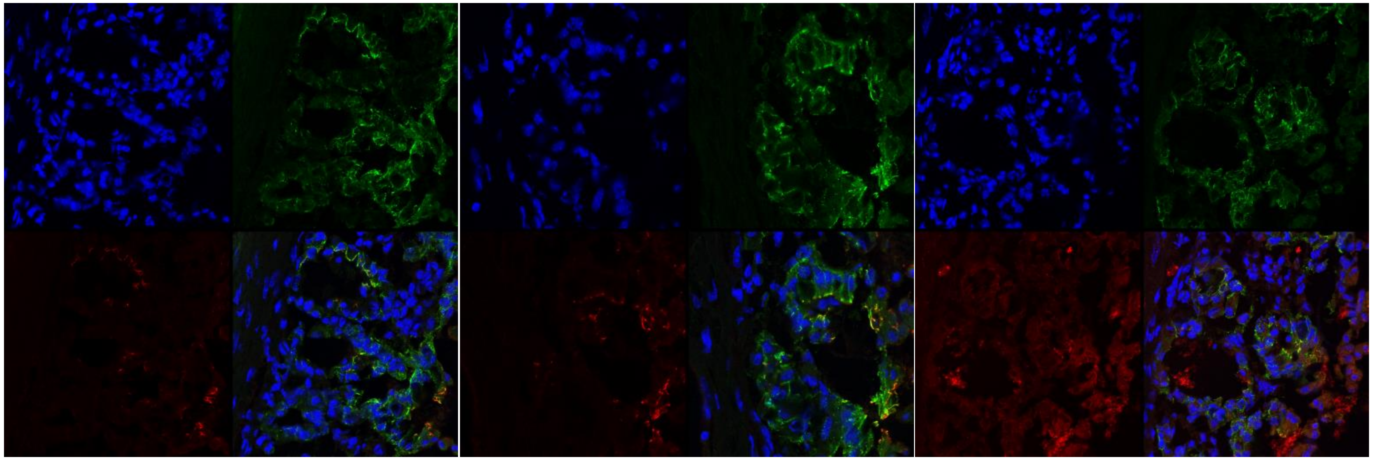

Figure S2. TJ dysregulation by DNBS. Colon from C57BL/6 mice were untreated (A) or exposed to DNBS (1,5mg/ml; B) for 5h, in GEVS. Tissue distribution of TJs was evaluated analyzing the localization of ZO-1 (red). Epithelial cells were evidenced by the expression of E-Cadherin (green), while cell nuclei were evidenced by DAPI (blue) staining.
